# Urinary Metabolomics in Bladder Cancer: A Scoping Review of Current Evidence and Translation Implications

**DOI:** 10.3390/ijms27208983

**Published:** 2026-10-10

**Authors:** Ângela Carapito, Filipa Amaro, Ana Cecília Roque, Félix Carvalho, Joana Pinto, Paula Guedes de Pinho

**Affiliations:** 1 Associate Laboratory i4HB—Institute for Health and Bioeconomy, University of Porto, 4050-313 Porto, Portugal; famaro@ff.up.pt (F.A.); felixdc@ff.up.pt (F.C.); jipinto@ff.up.pt (J.P.); 2 UCIBIO—Applied Molecular Biosciences Unit, Laboratory of Toxicology, Faculty of Pharmacy, University of Porto, 4050-313 Porto, Portugal; 3 Associate Laboratory i4HB—Institute for Health and Bioeconomy, NOVA School of Science and Technology, NOVA University of Lisbon, 2829-516 Caparica, Portugal; cecilia.roque@fct.unl.pt; 4 UCIBIO—Applied Molecular Biosciences Unit, Department of Chemistry, NOVA School of Science and Technology, NOVA University of Lisbon, 2829-516 Caparica, Portugal

**Keywords:** urinary metabolomics, biomarker discovery, bladder cancer, metabolic profiling, tumour stage and grade

## Abstract

Bladder cancer (BC) diagnosis and surveillance currently rely largely on invasive cystoscopy, making urine an attractive non-invasive biofluid for discovering metabolic biomarkers, given its direct contact with the bladder urothelium. This review provides a comprehensive and critical synthesis of the literature on urinary metabolomic profiling in BC, summarising metabolites and metabolite panels associated with the disease, their relevant metabolic pathways, and diagnostic performance. A search was conducted in PubMed, complemented by records from reference lists of relevant reviews. After screening, 30 original studies comparing urinary metabolomic profiles between BC patients and cancer-free controls were included, encompassing diverse analytical platforms and statistical approaches. A wide range of urinary metabolites were reported to be altered between BC patients and controls, of which 52 were recurrently identified in at least two independent studies. Increased levels of taurine, valine, lactate, adenosine monophosphate and adipate were among the most consistent metabolic alterations, whereas the levels of citrate, glycerol, creatinine, and several dicarboxylic acids and acylcarnitines were consistently decreased. Other metabolites, such as hippurate, tyrosine, and histidine, showed variable trends in their levels. These alterations may reflect metabolic processes associated with tumour growth, energy reprogramming, lipid metabolism, and oxidative stress adaptation. Most studies also proposed multi-metabolite panels combining metabolites to discriminate BC patients from controls, achieving sensitivity and specificity up to 100% and AUC up to 0.983, while fewer studies explored panels for tumour stage and grade discrimination, with more modest performance. Overall, urinary metabolomics reveals a broad, biologically coherent set of metabolic alterations associated with BC, but methodological heterogeneity and limited independent validation still hinder clinical translation. Larger, harmonised, prospectively validated studies integrating multi-omics and machine learning are needed to establish robust urinary biomarkers for BC.

## 1. Introduction

Bladder cancer (BC) is one of the most common types of cancer worldwide [1]. It represents a significant clinical and economic burden due to its high recurrence and the need for long-term surveillance, particularly depending on tumour stage and grade [2]. Although most patients are initially diagnosed with non-muscle-invasive BC (NMIBC), recurrence is common, and a substantial proportion of cases progress to muscle-invasive BC (MIBC), which is associated with poor prognosis and increased mortality [3]. Consequently, early diagnosis and accurate disease monitoring are essential to improve patient outcomes. Current diagnosis and surveillance of BC rely primarily on cystoscopy, urine cytology, imaging techniques, and histopathological examination [4]. However, these approaches have significant limitations. Cystoscopy, the diagnostic gold standard, is invasive and costly, whereas urine cytology has limited sensitivity for detecting low-grade disease [5]. These shortcomings have prompted the search for alternative and complementary diagnostic strategies, including the identification of tumour-associated proteins, nucleic acids, and chromosomal alterations in the urine of patients with BC [6]. However, none have demonstrated sufficient diagnostic accuracy, reproducibility, and clinical utility to replace cystoscopy in routine practice [6], which has fuelled the search for reliable, non-invasive biomarkers and innovative diagnostic approaches to improve BC management.

Metabolomics has emerged as a promising approach for identifying disease-specific biochemical alterations. As metabolic reprogramming is a hallmark of cancer, metabolomic profiling provides valuable insights into tumour biology while offering opportunities for biomarker discovery [7,8,9]. Among the available biological matrices, urine is particularly attractive because it can be collected non-invasively, is readily available in large volumes, and is in direct contact with the urothelium, making it especially suitable for BC research [10]. Advances in analytical platforms, particularly mass spectrometry (MS) and nuclear magnetic resonance (NMR) spectroscopy, have led to a growing number of metabolomic studies aimed at identifying urinary metabolic signatures capable of discriminating patients with BC from healthy individuals and signatures associated with tumour stage and grade [11,12]. These studies have consistently reported alterations in several metabolic pathways, including amino acid, energy, and lipid metabolism, as well as oxidative stress-related pathways. Despite these promising findings, the application of urinary metabolomics in clinical practice is limited by considerable heterogeneity across studies, hindering the identification of robust, reproducible biomarkers suitable for clinical use.

Although previous reviews have explored the application of metabolomics to BC biomarker discovery [11,12,13,14,15,16,17,18], the rapid expansion of urinary metabolomic studies and the emergence of novel biomarker candidates warrant an updated, comprehensive synthesis of the available evidence. Beyond summarising current knowledge, this review critically evaluates metabolic alterations consistently associated with BC and compiles urinary metabolite panels reported to discriminate patients with BC from healthy controls and those associated with tumour stage and grade. By identifying recurring metabolic signatures, this review aims to guide future metabolomic research and accelerate the development of clinically relevant urinary biomarkers for BC diagnosis and management.

## 2. Materials and Methods

### Literature Search and Study Selection

**Figure 1:**
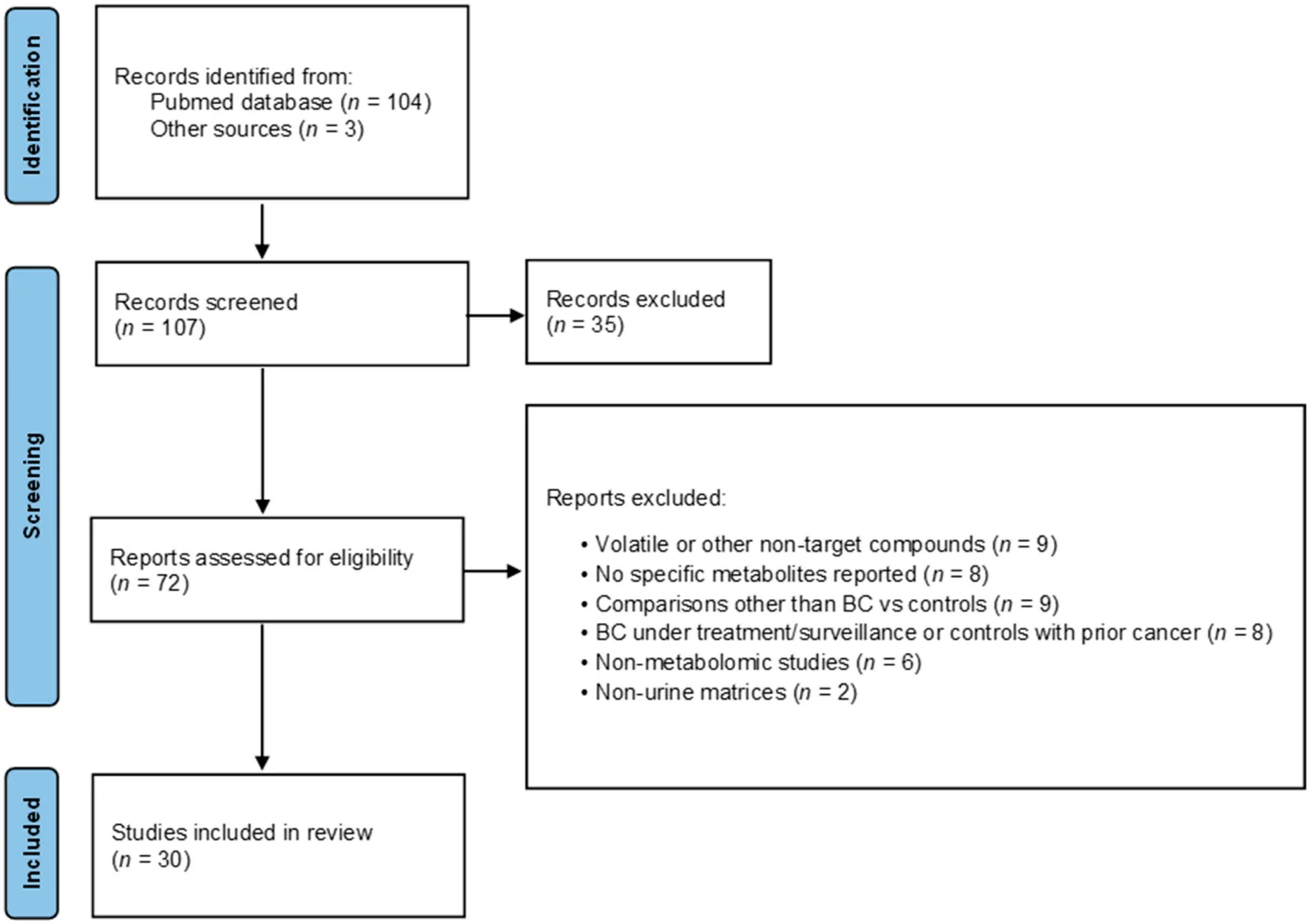
PRISMA flow diagram illustrating the identification, screening, and inclusion processes for studies considered in this review, focusing on metabolomics-based analyses of urinary metabolites for BC detection. The literature search covered articles published up to October 2025.

A literature search was conducted in PubMed in October 2025 to identify studies that applied metabolomic approaches to urine samples from BC patients and controls. The search terms used were (“(bladder cancer)” AND (metabolomics)) AND (urine), with no restriction on publication year and limited to English-language articles. The overall search strategy and study selection process are summarised in Figure 1. Original research articles investigating urinary metabolomic profiles in patients with BC were considered eligible. Studies unrelated to the scope of this review, non-original publications (e.g., reviews, conference abstracts, editorials, letters, and case reports), studies not involving human urine samples, and studies lacking sufficient methodological detail or robust statistical analyses were excluded. Two independent reviewers screened titles, abstracts, and full-text articles to determine study selection. The main information of each study, including cohort size and characteristics, analytical platform, statistical analysis, identified metabolites, direction of metabolite alteration, and, when available, performance metrics such as sensitivity, specificity, or area under the curve (AUC), was extracted. To facilitate the biological interpretation of the reported findings, significantly altered metabolites were annotated based on the Kyoto Encyclopedia of Genes and Genomes (KEGG) COMPOUND database [19] to identify their associated metabolic pathways.

## 3. Results and Discussion

### 3.1. Results

#### 3.1.1. Characteristics of Included Studies

The initial search (Figure 1) retrieved 104 studies from PubMed and three additional publications identified through the reference lists of relevant review articles. Of these, 35 were excluded as they were review papers, commentaries, study protocols, book chapters, or animal-based investigations. A second screening was then performed to ensure the inclusion of only original, human-based studies that directly compare urinary metabolomic profiles between BC patients and cancer-free controls. During this process, several articles were excluded for the following reasons: studies focused exclusively on volatile compounds or other analytical targets not relevant to this work (*n* = 9); studies that did not report or identify the specific metabolites detected, providing only general statistical outcomes or global metabolic trends (*n* = 8); studies that did not include BC as a disease group or that performed comparisons unrelated to BC versus controls (*n* = 9); studies involving BC patients under treatment or post-surgical surveillance, or those including control participants with a previous history of cancer (*n* = 8); studies that did not apply metabolomic techniques (*n* = 6); and studies analysing biological matrices other than urine, such as serum or tissue (*n* = 2). After applying these criteria, a total of 30 original research studies were selected for review, encompassing investigations that compared BC patients with cancer-free controls to identify disease-associated metabolic signatures. For clarity and consistency, the included studies and their main characteristics are presented in Table S1 in chronological order.

Overall, urinary metabolomic studies in BC (Table S1) exhibit substantial methodological diversity in study designs, cohort sizes, analytical platforms, and statistical approaches. Most investigations included fewer than 100 participants per group, reflecting the exploratory stage of this research field. Cohorts were generally divided into BC patients and cancer-free controls, although some studies also stratified patients according to tumour stage or grade to explore associations with disease progression. From an analytical perspective, the use of multiple analytical platforms provided complementary coverage of the urinary metabolome. GC–MS-based methods [20,21,22,23] remain widely applied due to their high chromatographic resolution, sensitivity, and reproducibility for detecting low-molecular-weight metabolites such as amino acids, organic acids, and fatty acid derivatives. LCMS-based approaches [6,24,25,26,27,28,29,30,31,32,33,34,35,36,37,38,39,40,41,42] broaden this coverage to include larger molecules, as well as more polar and nonpolar molecules, such as lipids, nucleosides, and peptides, and can operate under different separation modes, including reversed-phase (RPLC) and hydrophilic interaction (HILIC). In contrast, ^1^H NMR spectroscopy [43,44] was used in only two studies, making it particularly suitable for comparative profiling of abundant urinary metabolites. In addition to these platforms, several studies have investigated alternative or more advanced analytical techniques that broaden the scope of urinary metabolomics. For instance, high-performance liquid chromatography with fluorescence detection (HPLC-FLD) [45] enables the selective quantification of specific metabolite classes such as pterins. Meanwhile, matrix-assisted laser desorption/ionisation tandem time-of-flight mass spectrometry (MALDI–TOF/TOF–MS) and tip-contact extraction–laser desorption/ionisation mass spectrometry (TCE–LDI–MS) [46] have been developed as fast methods for analysing metabolites directly with very little sample preparation. Together, these techniques illustrate how metabolomic research continues to expand through different analytical approaches.

Data analysis procedures were generally consistent across studies, typically combining multivariate methods such as PCA, PLS-DA, and OPLS-DA to visualise clustering trends and identify discriminant metabolites. Univariate analyses, including *t*-tests, Mann–Whitney tests, or Kruskal–Wallis tests, were commonly used to assess differences in individual metabolites, often with false discovery rate (FDR) or Bonferroni corrections. Several recent studies [26,29,31,44] incorporated ML models such as Random Forest (RF), Support Vector Machine (SVM), or Classification and Regression Tree (CART), reflecting the growing use of machine learning methods in biomarker discovery. Validation strategies varied considerably. While many studies relied solely on internal cross-validation, others used independent validation cohorts, improving model robustness and reproducibility.

#### 3.1.2. Urinary Metabolic Alterations in BC Patients and Associated Pathways

Across the 30 studies included in this review, 317 distinct urinary metabolites were reported as significantly altered between BC patients and cancer-free controls. Of these, 52 were recurrently identified in at least two independent studies. These metabolites, summarised in Table 1, represent the most frequently reported urinary metabolic alterations associated with BC. The 52 recurrent metabolites were grouped according to their main putative metabolic class. Most were related to amino acid metabolism, which included 19 metabolites, followed by energy metabolism with 10 metabolites, lipid metabolism with four metabolites, nucleotide metabolism with five metabolites, and a group of 14 metabolites classified as “others”.

Among metabolites associated with amino acid metabolism, five were consistently reported to be elevated in BC patients: carnitine, proline, pyroglutamate, taurine, and valine. Taurine and valine were among the most frequently reported metabolites to be increased in this group. In contrast, carnitine C9:1, creatinine, pipecolate, and xanthurenate were consistently reported as decreased. The remaining amino acid-related metabolites showed inconsistent directions of change across studies, including 4-hydroxycinnamate, 5-hydroxyindoleacetic acid, carnitine C8:1, creatine, histidine, phenylacetylglutamine, S-adenosylmethionine, serine, tyrosine, and γ-aminobutyrate.

Within the energy metabolism group, lactate and erythritol were consistently elevated in BC patients, with lactate reported to be elevated in five independent studies. Conversely, citrate and glycolate were consistently decreased. Fructose, gluconate, N-acetylneuraminate, ribitol, and succinate showed both increased and decreased urinary levels across studies. Galactose was reported to be increased in one study, while another did not report a direction of change.

The lipid-related metabolites included adipate, glycerol, glycochenodeoxycholate, and O-phosphoethanolamine. Adipate and O-phosphoethanolamine were consistently increased, whereas glycerol was consistently decreased. Glycochenodeoxycholate showed inconsistent results, with both increased and decreased levels reported across studies.

Five recurrent metabolites were associated with nucleotide metabolism. AMP were consistently increased in BC patients, while adenosine was consistently decreased. Pseudouridine, urate, and uridine showed mixed directional changes. Uridine was among the most frequently reported metabolites in this category, with increases reported in five studies and decreases in one.

The remaining 14 metabolites were grouped as “others”. Most of these were consistently decreased, including 2,5-furandicarboxylate, 2,6-dimethylheptanoyl carnitine, azelate, decanoylcarnitine, ferulate, pyridoxate, ribonate, decanedioic acid, Sumiki’s acid, trigonelline, and trimethylxanthine. Niacinamide was the only metabolite in this group consistently reported as increased. Hippurate and nicotinurate showed inconsistent directions of change across studies. Hippurate was among the most frequently reported recurrent metabolites overall, with both increased and decreased urinary levels observed.

Overall, the recurrent urinary metabolites listed in Table 1 (reported in at least two independent studies) included 12 metabolites consistently increased in BC patients, 18 metabolites consistently decreased, 21 metabolites with inconsistent directions of change, and one metabolite (galactose) for which no clear tendency was reported in one study. These findings show that the urinary metabolic profile associated with BC encompasses a broad set of recurrent metabolites, including taurine, valine, lactate, citrate, uridine, hippurate, and several carnitine- or fatty acid-related compounds that appear across multiple independent studies.

#### 3.1.3. Urinary Metabolite Panels for Discriminating BC Patients from Controls

Since urinary metabolite panels are increasingly investigated in BC metabolomics, Table 2 summarises the panels identified across studies for discriminating BC patients from cancer-free controls. The table details the metabolite panel composition, the direction of metabolic alterations, and the diagnostic performance of each panel, including sensitivity, specificity, accuracy, and area under the curve (AUC).

Overall, 18 studies (Table 2) reported urinary metabolite panels for discriminating between BC patients and cancer-free controls. The number of metabolites included in the reported panels ranged from two [34] to 41 [37] metabolites. The diagnostic performance varied across studies and models, with sensitivity ranging from 71% to 100%, specificity ranging from 70% to 100%, accuracy values ranging from 83% to 100%, and AUC values ranging from 0.726 to 0.983. Some studies reported separate performance measures for training, validation, or test sets.

#### 3.1.4. Urinary Metabolite Panels for Discriminating BC Stage and Grade

Table 3 summarises the panels identified across the included studies for discriminating BC according to tumour stage and grade, providing information on the composition of each panel’s metabolites, the direction of metabolic alterations, the comparisons evaluated, and the reported diagnostic performance (sensitivity, specificity, accuracy and AUC).

Overall, seven studies reported urinary metabolite panels for distinguishing BC stage or grade. These panels were evaluated in various comparisons, including NMIBC vs. MIBC; NMIBC or MIBC vs. cancer-free controls; LG vs. HG disease; and LG or HG NMIBC vs. controls. The number of metabolites included in the reported panels ranged from two [42] to 27 [30]. Among the studies reporting each metric, the ranges were as follows: sensitivity, 63–91%; specificity, 67–97%; accuracy, 50–88%; and AUC, 0.739–0.954. Some studies reported these performance metrics separately for training or discovery sets and external validation sets.

### 3.2. Discussion

For the purpose of discussion, recurrent metabolites found in BC urine and listed in Table 1 were grouped into major metabolic pathways according to their biological context.

#### 3.2.1. Amino Acid Metabolism

Several recurrent urinary metabolites were linked to amino acid metabolism, highlighting the central role of amino acid-related biochemical alterations in cancer development and progression. Amino acids are essential for tumour growth and maintenance, acting as fundamental building blocks for protein synthesis, energy substrates, and precursors of antioxidants and signalling molecules. They also influence multiple biological processes, including cell proliferation, redox homeostasis, and epigenetic regulation, which contribute to tumour initiation, progression, and immune evasion [51,52].

Among the reported metabolites, taurine, valine, tyrosine, phenylacetylglutamine, and histidine were detected in at least 3 independent studies. Notably, taurine emerged as the most consistently elevated metabolite in BC patients compared with cancer-free controls [28,30,43,46,47]. As a sulphur-containing amino acid derived from cysteine, taurine contributes to osmotic regulation, bile acid conjugation, and cellular protection against oxidative stress [53]. Its urinary increase likely reflects a compensatory response to oxidative and inflammatory injury, as proposed by Li et al. [30] and Shi et al. [28], who suggested that enhanced taurine synthesis may promote cytoprotection while facilitating tumour persistence under stress conditions.

Valine, a branched-chain amino acid (BCAA), was also consistently elevated [21,24,41,46]. BCAAs supply both carbon and nitrogen to proliferating cells, serving as substrates for the tricarboxylic acid (TCA) cycle and as sources for nucleotide and lipid biosynthesis [54]. Its accumulation in urine reflects intensified protein and BCAA turnover in tumour cells, consistent with mitochondrial stress and altered anaplerotic flux [55]. In contrast, tyrosine, phenylacetylglutamine (PAGN), and histidine exhibited variable trends among studies.

Tyrosine was reported as both increased [25,41] and decreased [27,28]. As an aromatic amino acid derived from phenylalanine, tyrosine participates in several metabolic processes, including catecholamine and melanin biosynthesis [51]. However, its urinary excretion is also highly dependent on the efficiency of renal filtration and reabsorption. Alterations in renal function can substantially modify the clearance of tyrosine and phenylalanine, leading to apparent differences in urinary levels [56]. Therefore, the inconsistent findings among studies may reflect variable kidney handling of amino acids in BC patients rather than direct tumour-related metabolic changes. PAGN, a conjugate of phenylacetate and glutamine, was found to be decreased in several studies [28,42,47] but increased in another [25]. Shi et al. [28] suggested that reduced PAGN indicates dysregulation of phenylalanine and glutamine metabolism linked to enhanced energy demand and biosynthetic flux in tumour cells. Because PAGN is partly derived from gut microbial activity, variability in microbiota composition and nitrogen excretion likely contributes to the inconsistent results [57]. Histidine exhibited inconsistent behaviour, with some studies reporting elevated levels [41] and others reporting a reduction [30,33]. Łuczykowski et al. [33] proposed that decreased histidine may reflect diminished activity of the fragile histidine triad (FHIT) protein, a tumour suppressor frequently lost in HG BC, linking altered histidine metabolism to tumour aggressiveness and prognosis [58]. Differences in diet and systemic nitrogen balance may further contribute to this variability [59].

Altogether, the consistent elevation of taurine and valine points to a reprogramming of amino acids in BC, while the divergent patterns observed for tyrosine, PAGN, and histidine highlight the influence of oxidative status, tumour heterogeneity, and host–microbiome interactions.

#### 3.2.2. Energy Metabolism

Several recurrent urinary metabolites were associated with energy metabolism, including compounds involved in glycolysis, the TCA cycle, and carbohydrate oxidation. These compounds collectively reflect the metabolic adaptations that support tumour proliferation and survival. Cancer cells often rely on increased glycolytic flux and altered mitochondrial respiration to sustain rapid growth, a metabolic hallmark known as the Warburg effect, resulting in characteristic shifts in energy-related metabolites that can be traced in urine [60]. Among these metabolites, lactate, citrate, gluconate, and erythritol were the most reported, representing the most consistent indicators of disrupted energy metabolism in BC.

Among the most consistently altered metabolites, lactate was reported as increased in multiple studies [20,24,25,26,27]. Elevated lactate levels are a hallmark of the Warburg effect, in which tumour cells favour aerobic glycolysis even under normoxic conditions, leading to increased lactate production and excretion. This metabolic adaptation facilitates rapid adenosine triphosphate (ATP) generation, maintains redox balance, and contributes to an acidic tumour microenvironment that promotes invasion and immune evasion [61]. Similarly, citrate, a key TCA intermediate, was consistently found at lower levels in BC urine across several studies [20,28,43]. The reduction in urinary citrate may result from its increased cellular uptake to fuel lipid biosynthesis since intracellular citrate can be cleaved into acetyl coenzyme A (acetyl-CoA) and oxaloacetate by ATP-citrate lyase. Acetyl-CoA serves as a precursor for de novo fatty acid synthesis, a process essential for tumour proliferation. Additionally, impaired TCA cycle activity, along with enhanced β-oxidation and lipogenesis, may contribute to citrate depletion. Shi et al. [28] also proposed that this decrease could be linked to the Warburg effect, reflecting mitochondrial dysfunction and a shift toward glycolytic energy metabolism [60]. In contrast, gluconate showed inconsistent trends across studies, being reported as increased in three studies [24,35,36], but decreased in two [21,33]. None of the studies reporting elevated urinary levels provided a mechanistic explanation for this finding, although higher gluconate may reflect enhanced glucose oxidation and pentose phosphate pathway (PPP) activity, processes that supply nicotinamide adenine dinucleotide phosphate (NADPH) and biosynthetic precursors necessary for tumour growth and antioxidant defence [62]. In contrast, Łuczykowski et al. [33] suggested that reduced gluconic acid levels may result from its consumption under oxidative stress, given the reported antioxidant properties of gluconate and related derivatives. These contradictory findings are likely driven by variations in tumour stage, redox status, dietary intake, and methodological differences across studies, rather than a single underlying biological mechanism. Erythritol was consistently elevated in urine in three independent studies [20,23,25]. As a polyol produced via glucose reduction through the PPP, erythritol has been linked in vitro to increased carbohydrate flux and oxidative stress adaptation, since this pathway generates NADPH to support antioxidant defence and biosynthetic demands [63]. Elevated erythritol may therefore reflect a metabolic response to the heightened oxidative burden and anabolic demands characteristic of tumour proliferation [62].

#### 3.2.3. Lipid Metabolism

Although fewer in number, several metabolites associated with lipid metabolism were consistently reported across the reviewed studies, reflecting disruptions in fatty acid oxidation, membrane phospholipid turnover, and bile acid metabolism, processes closely linked to tumour growth and cellular homeostasis. Four metabolites, including adipate, glycerol, glycochenodeoxycholate, and O-phosphoethanolamine, were identified in at least two independent studies, highlighting the relevance of lipid-related pathways in BC metabolic reprogramming.

Adipate, a dicarboxylic acid formed through ω-oxidation of medium-chain fatty acids, was consistently elevated in the urine of BC patients [20,24]. This increase likely reflects enhanced fatty acid oxidation and peroxisomal activity, potentially as a metabolic adaptation to meet the heightened energy demands and oxidative stress associated with tumour cells [64]. The accumulation of dicarboxylic acids occurs systemically, particularly in tissues active in fatty acid metabolism, and is reflected in increased urinary excretion. This systemic elevation has been associated with mitochondrial dysfunction and compensatory activation of alternative lipid-degradation pathways, both recognised hallmarks of altered cancer metabolism [65]. In contrast, glycerol was consistently decreased in urine of BC patients in two studies [20,21]. Pasikanti et al. [21] suggested that this reduction reflects increased glycerol utilization for gluconeogenesis, driven by the high glycolytic activity of tumour cells. Hepatic conversion of glycerol into glucose may thus serve as an adaptive mechanism to meet the elevated energy and biosynthetic demands of proliferating tumour cells [66]. Thus, lower urinary glycerol likely reflects a systemic metabolic shift favouring glucose production and energy supply for tumour progression. O-Phosphoethanolamine, an intermediate in glycerophospholipid biosynthesis, was also reported as elevated in BC urine [34,39]. This metabolite is a precursor of phosphatidylethanolamine, a key component of cellular membranes, and plays an important role in cell proliferation, membrane remodelling, and lipid signalling [67]. Chen et al. [34] suggested that elevated levels of phosphatidylethanolamine may reflect increased membrane turnover and oncogenic signalling associated with rapid tumour cell growth, a pattern consistent with the general observation of enhanced phospholipid metabolism in malignancy. Finally, glycochenodeoxycholate, a bile acid conjugate, exhibited divergent trends, being increased in Wang et al. [26] but decreased in Guo et al. [27]. Wang et al. [26] interpreted the observed increase as indicative of dysregulated bile acid metabolism, which may contribute to bladder mucosal irritation and carcinogenesis via cytotoxic, oxidative, and inflammatory mechanisms. Conjugated bile acids, such as glycochenodeoxycholate, have been shown to activate intracellular signalling pathways, including extracellular signal-regulated kinase (ERK1/2) and AKT, thereby promoting tumour cell proliferation and invasiveness [68]. In contrast, the decreased levels reported by Guo et al. [27] may be attributable to inter-individual variations in dietary intake, hepatic function, or tumour stage, factors known to modulate bile acid biosynthesis, conjugation, and excretion.

#### 3.2.4. Nucleotide Metabolism

Alterations in nucleotide metabolism have been consistently reported across multiple studies, reflecting the elevated biosynthetic requirements and accelerated RNA turnover that typify cancer cells. Within this metabolic pathway, adenosine, adenosine monophosphate (AMP), pseudouridine, urate, and uridine were identified, with pseudouridine and uridine emerging as the most frequently reported biomarkers.

Both pseudouridine and uridine were detected as significantly altered metabolites in the urine of patients with BC. Pasikanti et al. [20] and Yumba-Mpanga et al. [36] reported elevated urinary levels of both metabolites in BC patients compared with controls, whereas Shi et al. [28] observed decreased pseudouridine levels. Pasikanti et al. [20] interpreted this elevation as indicative of hyperactive RNA metabolism, consistent with the heightened energetic and biosynthetic demands of tumour cells. Similarly, Yumba-Mpanga et al. [36] proposed that pseudouridine, a post-transcriptionally modified nucleoside generated through RNA isomerization, is released during RNA degradation and excreted in urine without further modification. Consequently, increased urinary pseudouridine may reflect enhanced RNA turnover and diminished activity of pseudouridine kinase, the enzyme responsible for its reconversion to pseudouridine-5′-phosphate. Uridine, a key pyrimidine nucleoside and essential precursor in RNA synthesis, showed a general upward trend across most studies [20,21,25,34,36], with only Peng et al. [39] reporting decreased levels. These increases likely reflected enhanced nucleotide biosynthesis and degradation associated with the rapid cell proliferation characteristic of tumour metabolism. Yumba-Mpanga et al. [36] further suggested that elevated uridine levels may result from dysregulated activity of uridine nucleosidase and uridine phosphorylase, enzymes responsible for converting uridine to uracil. In contrast, adenosine and AMP were less frequently reported but displayed consistent trends across studies: adenosine levels were decreased in two reports [24,30], whereas AMP [30,33] concentrations were consistently elevated.

#### 3.2.5. Other Metabolites

The metabolites categorized as “others” constitute a small but chemically diverse group of compounds recurrently reported across studies. They do not clearly align with amino acid, energy, nucleotide, or lipid metabolism pathways, and often lack annotation in the KEGG [19]. Despite their heterogeneity, many of these metabolites may be biologically significant, reflecting systemic physiological changes or exogenous influences on urinary metabolic profiles.

A first subgroup includes dicarboxylic acids and acylcarnitines, such as azelate (nonanedioic acid), sebacic acid (decanedioic acid), 2,6-dimethylheptanoyl carnitine, and decanoylcarnitine, which are intermediates of fatty acid ω-oxidation and mitochondrial β-oxidation [65]. These metabolites often indicate altered lipid catabolism or peroxisomal dysfunction, metabolic alterations frequently associated with oxidative stress and redox imbalance in cancer [69]. A second subgroup encompasses aromatic and phenolic derivatives, including ferulate, hippurate, trigonelline, 5-hydroxymethyl-2-furoate (Sumiki’s acid), and 2,5-furandicarboxylate. Many of these compounds arise from dietary sources, polyphenol metabolism, or gut microbiota co-metabolism, linking the urinary metabolome to diet-microbiome interactions that influence host inflammation and carcinogenesis [70]. A third subgroup consists of vitamin- and cofactor-related metabolites, namely niacinamide, nicotinurate, and pyridoxate, which are associated with vitamin B_3_ and B_6_ metabolic pathways. These metabolites play key roles in maintaining redox and cofactor homeostasis, particularly the NAD^+^/NADP^+^ balance, whose disruption can compromise oxidative stress regulation and DNA repair mechanisms in tumour cells [71]. Finally, trimethylxanthine (caffeine), despite being primarily of exogenous origin, was reported in two studies [27,35] to be decreased in the urine of BC patients. This reduction may reflect differences in dietary intake, hepatic metabolism, or renal excretion.

Among this heterogeneous group of metabolites, hippurate emerged as the most consistently reported compound, being identified in several studies with both decreased [25,33,36,42,43,47] and increased [28,41] urinary levels. Hippurate, a conjugate of benzoic acid and glycine, is a normal urinary constituent synthetized mainly in the liver and kidneys, and its levels are strongly influenced by gut microbial activity and dietary intake. It is involved in phenylalanine, glycine, and microbial metabolic pathways [72]. Luczykowski et al. [33] and Yumba Mpanga et al. [36] suggested that the reduced hippurate levels may result from altered gut microbiota activity or impaired hepatic conjugation capacity, potentially involving changes in the activity of enzymes associated with hippurate metabolism. Similarly, Jacyna et al. [25] proposed that lower hippurate levels reflect microbial dysbiosis and diminished metabolism of aromatic compounds. In contrast, Srivastava et al. [43] emphasized that such variations could also be influenced by dietary benzoate intake or toluene exposure, highlighting the multifactorial nature of hippurate fluctuations. Conversely, Shi et al. [28] and Putluri et al. [41] reported elevated urinary hippurate levels, which may reflect compensatory detoxification processes or renal excretory adaptations in response to tumour-induced metabolic stress. Furthermore, nicotinurate demonstrated divergent trends across studies, being increased in Shen et al. [38] and decreased in Yumba Mpanga et al. [35]. As a downstream product of nicotinate and nicotinamide metabolism and a minor derivative of fatty-acid β-oxidation, nicotinurate reflects NAD^+^-dependent redox and energy metabolism. Elevated levels may denote enhanced nicotinamide turnover and oxidative stress, whereas reduced concentrations could signify impaired vitamin B_3_ utilization or renal reabsorption [73].

#### 3.2.6. Urinary Metabolite Panels for BC Diagnosis, Stage and Grade

Urinary metabolite panels offer a more comprehensive representation of the metabolic alterations associated with BC than individual metabolites by integrating changes involved in different biochemical pathways. Across the reviewed studies, panel composition varied considerably, reflecting differences in study populations, analytical platforms, and statistical approaches. Despite this heterogeneity, investigations incorporating both internal and external validation [20,21,22,23,39,44,49] provided stronger evidence of model performance, emphasising the robustness and potential clinical reliability of metabolite-based diagnostic models when tested across independent cohorts.

For BC detection (BC vs. cancer-free individuals, Table 2), metabolites that were recurrently identified across these panels included intermediates of energy metabolism (e.g., lactate [20,24], citrate [20,21], succinate [20,40], gluconate [20,21,24]), amino acid metabolism (e.g., valine [21,37,41], histidine [30,41]), and nucleotide metabolism (e.g., uridine [20,21,34,39]), aligning with the central metabolic disturbances previously described. Several studies also identified lipid-related molecules such as O-phosphoethanolamine [34,39], adipate [20], and glycochenodeoxycholate [26], alongside with microbial-derived compounds like hippurate [41,44], further highlighting the interplay between host metabolism and microbiome in shaping the urinary metabolic phenotype of BC. Notably, several models achieved excellent diagnostic performance (AUC ≥ 0.95) with relatively small metabolite subsets [26,28,30], reinforcing the potential of streamlined urinary panels as practical, non-invasive tools for BC detection.

Beyond diagnosis, urinary metabolomics has also been investigated for the discrimination of tumour stage and grade (Table 3). The available evidence suggests that urinary metabolic profiles can capture differences associated with tumour aggressiveness, particularly when comparing NMIBC with MIBC and LG with HG disease. However, the metabolic signatures identified were not consistently reproduced across studies. Only hippurate was consistently identified in two independent studies [36,44], with both reporting decreased urinary levels in HG disease, suggesting reduced microbial co-metabolism or impaired aromatic compound clearance. Although myristic acid was also detected by Wang et al. [26] and Cheng et al. [50], the groups compared differed: Wang et al. [26] observed decreased levels in HG vs. LG BC, whereas Cheng et al. [50] found increased levels in LG NMIBC vs. controls. Therefore, this metabolite does not exhibit consistent recurrence across independent datasets.

An additional observation is that some studies evaluated diagnostic panels for stage or grade classification rather than developing dedicated signatures. Although this suggests that metabolic alterations associated with BC detection may also capture aspects of tumour aggressiveness, the evidence for specific stage- or grade-related signatures remains limited. Overall, urinary metabolomics shows promise for extending BC assessment beyond diagnosis, but larger cohorts, harmonised analytical workflows, and independent external validation are required to establish clinically robust signatures for tumour stage and grade.

#### 3.2.7. Challenges for the Clinical Translation of Urinary Metabolomics

Urinary metabolomics has demonstrated considerable potential for the discovery of non-invasive biomarkers for BC. Over the past decade, numerous studies have identified urinary metabolite panels with encouraging diagnostic performance, demonstrating the ability to discriminate patients with BC from healthy controls and, in some cases, to distinguish tumour stage and grade. Collectively, these findings highlight the considerable potential of urinary metabolomics to improve BC diagnosis, risk stratification, and disease monitoring. However, despite these promising results, only a limited number of metabolite panels have been successfully validated in independent cohorts. Differences in analytical platforms, sample preparation, metabolite identification, statistical strategies, and cohort characteristics continue to limit comparability across studies and hinder the clinical translation of metabolomic findings. Furthermore, urinary metabolic profiles may be influenced by biological and clinical factors, including comorbidities, environmental exposures, lifestyle factors, and previous treatments, introducing additional variability into urine-based analyses. Consequently, the identification of robust, reproducible, and clinically applicable biomarkers capable of accurately discriminating BC, stratifying tumour aggressiveness, detecting disease progression, and predicting recurrence remains a major challenge [74,75].

Future research should follow a structured path from metabolomic discovery to clinical translation. In the discovery stage, complementary analytical platforms may be combined to increase metabolic coverage, followed by targeted quantification of candidate metabolites in adequately powered, independent cohorts. The modelling stage should use machine learning approaches with nested cross-validation, rigorous prevention of data leakage and overfitting, and external validation. Subsequent prospective, multicentre, and registered studies should use standardized reference standards and quality-control procedures to evaluate validated metabolomic signatures against or in combination with established diagnostic approaches. Finally, regulatory requirements and cost-effectiveness should be considered to support the translation of promising metabolomic signatures into clinical practice. This stepwise approach will be essential to determine whether urinary metabolomic signatures can provide robust and clinically meaningful diagnostic tools for BC and support their integration into precision medicine.

#### 3.2.8. Limitations and Considerations of the Review

Some limitations should be considered when interpreting the findings of this review. Firstly, this work was designed as a scoping review to map and synthesise the available evidence on urinary metabolomic alterations in BC; therefore, a formal assessment of methodological quality or risk of bias, as is typically performed in systematic reviews, was not conducted. Data extraction focused on metabolomic findings, analytical approaches and reported associations with BC diagnosis, tumour stage and grade. Other clinical, demographic and pre-analytical variables were not systematically extracted and some potential covariates and confounding factors were not considered or reported in the original studies. Notably, fasting status, hydration, sampling time and urine collection and processing procedures were not consistently reported or standardised, which may contribute to variability in urinary metabolic profiles. This is particularly relevant for metabolites such as taurine, hippurate, and phenylacetylglutamine (PAGN), which are influenced by factors including gut microbiota, renal function, and dietary intake. Therefore, their potential use as diagnostic biomarkers should be approached with caution, as some metabolic alterations may reflect the host’s broader metabolic state rather than tumour-specific mechanisms. Similarly, the diagnostic performance reported by some studies should be considered with careful consideration, as several models were developed using relatively small cohorts and lacked independent external validation, which increases the potential for overfitting and optimistic performance estimates. Nevertheless, this review provides a structured synthesis of urinary metabolites reported across studies and identifies recurrent metabolic alterations that may inform and refine future research aimed at validating clinically relevant BC biomarkers.

## 4. Conclusions

This review systematises the available evidence on the urinary metabolome associated with BC, bringing together 30 original studies comparing BC patients with cancer-free controls. Fifty-two metabolites were recurrently reported, constituting a set of candidate urinary biomarkers. These metabolites were mainly distributed across amino acid, energy, lipid, and nucleotide metabolism pathways, reflecting metabolic alterations widely recognised as hallmarks of the tumour phenotype, namely the Warburg effect, increased de novo fatty acid synthesis, intensified protein and nucleotide turnover, and an adaptive response to oxidative stress.

Among the most consistent findings, elevations in urinary taurine, valine, lactate, AMP and adipate stood out, alongside decreases in citrate, glycerol, creatinine, and several dicarboxylic acids and acylcarnitines. In contrast, metabolites such as hippurate, tyrosine, histidine, and phenylacetylglutamine showed inconsistent trends across studies, likely reflecting the influence of confounding factors such as diet, gut microbiota, renal function, and tumour heterogeneity.

Beyond identifying individual metabolites, most of the reviewed studies proposed multi-metabolite panels with generally high diagnostic performance, highlighting the potential of urinary metabolomics as a non-invasive tool for BC detection. A smaller number of studies further explored the ability of these panels to discriminate tumour stage and grade, with promising but less consistent and less validated results.

Despite these advances, the clinical translation of urinary metabolomics in BC remains limited by methodological heterogeneity across studies, namely in analytical platforms, sample size, patient selection criteria, and statistical strategies, as well as by the scarcity of validation in independent, multicentre cohorts. Future studies, ideally prospective, with harmonised protocols and the integration of multiomics and machine learning approaches, will be essential to consolidate robust and clinically applicable urinary metabolic panels that support early diagnosis, risk stratification, and monitoring of bladder cancer progression.

## Figures and Tables

**Table 1:** Urinary metabolites reported in at least two independent studies as significantly altered between BC patients and controls, with corresponding metabolic pathways and direction of change.

Metabolite	Up-/Down-Regulated [Ref] ^a^	Putative Metabolic Pathways ^b^
**Amino acid metabolism**		
4-Hydroxycinnamate	↑ [20]; ↓ [33]	Tyrosine metabolism
5-Hydroxyindoleacetic acid (5-HIAA)	↑ [39]; ↓ [33]	Tryptophan metabolism
Carnitine	↑ [40,41]	Lysine degradation
Carnitine C8:1	↑ [47]; ↓ [21]	Lysine degradation
Carnitine C9:1	↓ [42,47]	Lysine degradation
Creatine	↑ [41]; ↓ [24]	Glycine, serine and threonine metabolism; arginine and proline metabolism
Creatinine	↓ [28,46]	Arginine and proline metabolism
Histidine/L-Histidine	↑ [41]; ↓ [30,33]	Histidine metabolism; β-alanine metabolism; aminoacyl-tRNA biosynthesis; protein digestion and absorption
Phenylacetylglutamine (PAGN)	↑ [25]; ↓ [28,42,47]	Phenylalanine metabolism
Pipecolate	↓ [25,41]	Lysine degradation
Proline	↑ [26,46]	Arginine and proline metabolism; D-amino acid metabolism; aminoacyl-tRNA biosynthesis; protein digestion and absorption
Pyroglutamate	↑ [39,48]	Glutathione metabolism
S-Adenosylmethionine (SAM)	↑ [41]; ↓ [25]	Cysteine and methionine metabolism; arginine and proline metabolism; one carbon pool by folate; sulfur relay system
Serine	↑ [41]; ↓ [27]	Glycine, serine and threonine metabolism; cysteine and methionine metabolism; glycerophospholipid metabolism; sphingolipid metabolism; glyoxylate and dicarboxylate metabolism; one carbon pool by folate; aminoacyl-tRNA biosynthesis; carbon metabolism
Taurine	↑ [28,30,43,46,47]	Primary bile acid biosynthesis; taurine and hypotaurine metabolism
Tyrosine	↑ [25,41]; ↓ [27,28]	Tyrosine metabolism; phenylalanine metabolism; phenylalanine, tyrosine and tryptophan biosynthesis; thiamine metabolism; 2-oxocarboxylic acid metabolism
Valine	↑ [21,24,41,46]	Valine, leucine and isoleucine degradation; valine, leucine and isoleucine biosynthesis; pantothenate and CoA biosynthesis; 2-oxocarboxylic acid metabolism
Xanthurenate	↓ [24,27]	Tryptophan metabolism
γ-Aminobutyrate (GABA)	↑ [46]; ↓ [24]	Alanine, aspartate and glutamate metabolism; arginine and proline metabolism; butanoate metabolism
**Energy metabolism**		
Citrate	↓ [20,28,43]	Citrate cycle (TCA cycle); alanine, aspartate and glutamate metabolism; glyoxylate and dicarboxylate metabolism; carbon metabolism
Erythritol (meso-erythritol)	↑ [20,23,25]	-
Fructose	↑ [23]; ↓ [21,24]	Fructose and mannose metabolism; galactose metabolism; starch and sucrose metabolism; amino sugar and nucleotide sugar metabolism; carbohydrate digestion and absorption
Galactose	↑ [23]; * [32]	Galactose metabolism; carbohydrate digestion and absorption
Gluconate	↑ [24,35,36]; ↓ [21,33]	Pentose phosphate pathway
Glycolate	↓ [22,25]	Glyoxylate and dicarboxylate metabolism
Lactate	↑ [20,24,25,26,27]	Glycolysis/Gluconeogenesis; fructose and mannose metabolism; pyruvate metabolism; propanoate metabolism
N-Acetylneuraminate	↑ [36]; ↓ [27]	Biosynthesis of various nucleotide sugars
Ribitol	↑ [23]; ↓ [20,21]	Pentose and glucuronate interconversions; riboflavin metabolism
Succinate	↑ [40]; ↓ [24]	Citrate cycle (TCA cycle); oxidative phosphorylation; alanine, aspartate and glutamate metabolism; lysine degradation; tyrosine metabolism; phenylalanine metabolism; pyruvate metabolism; glyoxylate and dicarboxylate metabolism; propanoate metabolism; butanoate metabolism; nicotinate and nicotinamide metabolism; carbon metabolism
**Lipid metabolism**		
Adipate	↑ [20,24]	Fatty acid degradation (via ω-oxidation)
Glycerol	↓ [20,21]	Galactose metabolism; glycerolipid metabolism
Glycochenodeoxycholate	↑ [26]; ↓ [27]	Primary bile acid biosynthesis; secondary bile acid biosynthesis; bile secretion; cholesterol metabolism
O-Phosphoethanolamine	↑ [34,39]	Glycerophospholipid metabolism; sphingolipid metabolism
**Nucleotide metabolism**		
Adenosine	↓ [24,30]	Purine metabolism; one-carbon pool by folate; nucleotide metabolism
Adenosine monophosphate (AMP)	↑ [30,33]	Purine metabolism; nucleotide metabolism; biosynthesis of cofactors
Pseudouridine	↑ [20,36]; ↓ [28]	Pyrimidine metabolism
Urate	↑ [25]; ↓ [28]	Purine metabolism; bile secretion
Uridine	↑ [20,21,25,34,36]; ↓ [39]	Pyrimidine metabolism; nucleotide metabolism
**Others**		
2,5-Furandicarboxylate	↓ [20,21]	-
2,6-Dimethylheptanoyl carnitine	↓ [42,49]	Fatty acid degradation
Azelate (nonanedioic acid)	↓ [28,29]	Fatty acid degradation
Decanoylcarnitine	↓ [28,40]	Fatty acid degradation
Ferulate	↓ [27,36]	-
Hippurate	↑ [28,41]; ↓ [25,33,36,42,43,47]	-
Niacinamide	↑ [30,41]	-
Nicotinurate	↑ [38]; ↓ [35]	Nicotinate and nicotinamide metabolism
Pyridoxate	↓ [24,41]	Vitamin B6 metabolism
Ribonate	↓ [20,21]	-
Decanedioic acid (sebacic acid)	↓ [28,30]	Fatty acid degradation
Sumiki’s acid (5-Hydroxymethyl-2-furoate)	↓ [20,21]	-
Trigonelline	↓ [42,44]	-
Trimethylxanthine (caffeine)	↓ [27,35]	-

^a^ Arrows indicate direction of change, with ↑ representing increased levels and ↓ representing decreased levels, respectively. ^b^ Metabolic pathways were assessed using the KEGG Homo sapiens pathway library. * No tendency reported. TCA, tricarboxylic acid.

**Table 2:** Urinary metabolite panels reported in metabolomics studies as significantly discriminating BC patients from controls, with corresponding direction of change and performance metrics.

Study, Year	Panel Composition (Trend/Metabolite Name)	Performance Metrics
Shi et al., 2025 [28]	↓ Indoxyl sulfate; urate; 4-androstene-3α,17β-diol	Sens = 90% Spec = 100% AUC = 0.983
Yang et al., 2023 [49]	↑ 5-Methyldeoxycytidine ↓ Deoxyinosine; 2,6-dimethylheptanoyl carnitine; prostaglandin I2	Training: Sens = 91% Spec = 71% AUC = 0.875 Validation: Sens = 78% Spec = 84% AUC = 0.845
Niziol et al., 2023 [29]	↑ Oleamide; N-α-acetyllysine ↓ Azelate; isostearic acid; indoleacetic acid	AUCs > 0.979 Spec/Sens > 80%/81%
Ossolinski et al., 2023 [44]	↓ Trigonelline; urea; hippurate; 4-hydroxyphenylacetate; mannitol	Training: Sens = 87% Spec = 79% Accu = 83% AUC = 0.828 Validation: Sens = 87% Spec = 80% Accu = 83% AUC = 0.828
Li et al., 2022 [30]	↑ Isoleucyl-phenylalanine; choline; 1-methylhistidine; N-undecanoylglycine; linoleyl carnitine; adenosine monophosphate (AMP); niacinamide; phenylalanyl-asparagine; taurine ↓ Mesobilirubinogen; 2-hydroxycaproic acid; L-urobilin; 6-Hydroxyhexanoic acid; cytidine; dihydrotestosterone; L-histidine; flavin mononucleotide; valyl-serine; 4-acetamidobutanoic acid; (5R)-5-hydroxyhexanoic acid; adenosine; L-targinine; 1,11-undeanedicarboxylic acid; ubiquinone-2; butenylcarnitine; sebacic acid; alanyl-tyrosine	Sens = 91% Spec = 87% Accu = 89% AUC = 0.951
Wang et al., 2022 [26]	↑ AMP; proline; chenodeoxycholate; glycochenodeoxycholate ↓ Hydroxypropionic acid; picolinic acid; 4-hydroxybenzoic acid; salicyluric acid; N-acetylserine; 5-aminolevulinic acid; myristic acid	Sens = 95% Spec = 100% AUC = 0.983
Lin et al., 2021 [23]	↑ Desaminotyrosine; erythritol; D-ribose; ribitol; D-frutose; D-mannose; D-galactose	Training: AUC = 0.976 Test: AUC = 0.906
Chen et al., 2019 [34]	↑ O-Phosphoethanolamine; uridine	AUC = 0.726
Liu et al., 2018 [37]	↑ 4-Ethyl-2-methoxyphenol; 15-deacetylcalonectrin; crosatoside B; 9-decenoylcarnitine; 1-5-amino-5-carboxypentylamino-1-deoxyfructose; 4,5-dihydro-drospirenone-3-sulfate; O-desmethyl-tramadoglucuronide; 3-methyl-3-heptene; 3,8-dihydroxy-6-methoxy-7,11-eremophilen-12,8-olide; N-jasmonoyltyrosine; glutamyl-arginine; 20-trihydroxy-leukotriene-B4; hydroxyhexanoycarnitine; serinyl-valine; niazirin; 2-(1-ethoxyethoxy)propanoic acid; saxitoxin; 3-hydroxynonanoyl carnitine; minaprine; budesonide ↓ trans-2-Dodecenoylcarnitine; α-N-phenylacetyl-L-glutamine; pyrogallol-2-O-glucuronide; diethylcarbamazine N-oxide; feruloyl-2-hydroxyputrescine; theogallin; 2E,4E-2,4-hexadienoic acid; L-pyridosine; 7-methylxanthine; S-N-4,5-dihydro-1-methyl-4-oxo-1H-imidazol-2-ylalanine; 6-amino-5N-methylformylamino-1-methyluracil; 5,5′,6,6′-tetrahydroxy-3,3′-biindolyl; 2-amino-4-ethoxy-3-hydroxybutanoic acid	Training: Sens = 88% Spec = 88% AUC = 0.956
Zhou et al., 2017 [22]	↑ Cholesterol ↓ 5-Hydroxyvaleric acid; 3-phosphoglycerate; glycolate	Training: Sens = 88% Spec = 86% AUC = 0.885 Validation: Sens = 78% Spec = 70% AUC = 0.804
Shen et al., 2015 [38]	MixModel 1: ↑ Nicotinuric acid; trehalose; AspAspGlyTrp ↓ Inosinate; ureidosuccinic acid; GlyCysAlaLys MixModel 2: ↑ AspAspGlyTrp ↓ Ureidosuccinic acid; GlyCysAlaLys	MixModel 1: Sens = 91% Spec = 81% AUC = 0.934 MixModel 2: Sens = 83% Spec = 90% AUC = 0.919
Wittmann et al., 2014 [24]	↑ Lactate; palmitoyl sphingomyelin; gluconate ↓ 2-Methylbutyrylglycine; guanidinoacetate; adenosine	Cohort 1: AUC = 0.81 Cohort 2: AUC = 0.78
Peng et al., 2014 [39]	↑ 3-Amino-2-piperidone; pyroglutamic acid; O-phosphoethanolamine; 5-hydroxyindoleacetic acid ↓ Dihydrolipoate; uridine; N-acetylputrescine + C2H4; histamine + C2H3NO; L-prolyl-L-proline + NH	Training: AUC = 0.956 Verification: AUC = 0.935
Jin et al., 2014 [40]	↑ Succinate; pyruvate; oxoglutarate; carnitine; phosphoenolpyruvate; trimethyllysine; isovalerylcarnitine; octenoylcarnitine; acetyl-CoA ↓ Melatonin; glutarylcarnitine; decanoylcarnitine	PLS-DA (ROC): Sens = 85% Spec = 85% AUC = 0.937 OPLS-DA: Sens = 91% Spec = 92%
Pasikanti et al., 2013 [20]	↑ Adipate; anthranilic acid; coumaric acid derivative; cyclopentane-1,2-diamine; erythritol; erythronic acid; ethylmalonic acid; gluconic acid derivative; heptadecanoic acid; hydroxybutyric acid; itaconic acid; lactate; melibiose; N-acetylanthranilic acid; p-cresol; pseudouridine; talonic acid; uridine; vanillylmandelic acid; 2,3,4,5-tetrahydroxypentanoic acid-1,4-lactone; 2-aminoisobutyric acid; 2-hydroxymalonic acid; 2-pentadecanol; 3,4-dihydroxyphenylpyruvate; 3-hydroxysebacic acid; 3-methyladipic acid; 4-methoxycinnamic acid ↓ Citrate; dihydroxyacetone; ethyl tartrate; gluconate; glycerol; levulinic acid; pinene; ribitol; ribonate; sebacic acid; sumiki’s acid; 2,5-furandicarboxylic acid; succinate; 2-hydroxyglutaric acid	Training: Sens/Spec = 100% AUC = 0.88 Validation: Sens = 71% Spec = 100%
Huang et al., 2011 [42]	↑ Component I (2-(1-(7-(butyl(ethyl)- amino)heptyloxy)propan-2-yloxy)ethanol ↓ Carnitine C9:1	Sens = 93% Spec = 97% AUC = 0.963
Putluri et al., 2011 [41]	↑ Leucine/isoleucine; histidine; kynurenine; hypoxanthine; valine; phenylalanine; 3−hydroxykynurenine; tyrosine; carnitine; spermidine; lysine; serine; N12−acetylspermidine; hippurate; niacinamide; S−adenosylmethionine; thymine; D−ribonolactone; guanine; uracil; creatine ↓ 4−Pyridoxic acid; pipecolate; histamine; palmitic acid	Group 1 AUC = 0.79 Group 4 AUC = 0.76
Pasikanti et al., 2010 [21]	↑ Melibiose; uridine; L-valine ↓ Senecic acid; 2-butenedioic acid; ribonate; 2,5-furandicarboxylic acid; Sumiki’s acid; 2-propenoic acid; glycerol; gluconate; valerate; frutose; citrate; ribitol	Training: Sens/Spec = 100% AUC = 0.90 Validation: Sens = 92% Spec = 80%

Arrows indicate the direction of metabolite change in the urine of BC patients compared with controls (↑ = higher in BC; ↓ = lower in BC). Accu, accuracy; AMP, adenosine monophosphate; AUC, area under the curve; BC, bladder cancer; Ct, controls; Sens, sensitivity; Spec, specificity. Information on the dataset or validation approach is reported when available in the original study.

**Table 3:** Summary of urinary metabolite panels identified in metabolomics studies that significantly differentiate BC stages or grades, including metabolite composition, direction of change, and diagnostic performance metrics.

Study, Year	Panel Composition (Trend/Metabolite Name)	Performance Metrics
Shi et al., 2025 [28]	↓ γ-Glutamylleucine; L-octanoylcarnitine; heptanoylcarnitine	MIBC vs. NMIBC Sens = 90% Spec = 90% AUC = 0.941
Niziol et al., 2023 [29]	HG vs. Ct: ↑ Oleamide; n-α-acetyllysine ↓ Azelate; isostearic acid; indoleacetic acid; 2-furoylglycine; cis, cis-muconic acid; phenylglyoxylic acid; methylmalonic acid; 3,4-dihydroxymandelic acid; n-acetylserotonin; 3-hydroxy-4-methoxycinnamic acid Triglyglycine LG vs. Ct: ↓ Isostearic acid; indoleacetic acid	HG/LG vs. Ct AUCs > 0.900 Spec/Sens > 87%/80%
Li et al., 2022 [30]	↑ Isoleucyl-phenylalanine; choline; 1-methylhistidine; n-undecanoylglycine; linoleyl carnitine; adenosine monophosphate (AMP); niacinamide; phenylalanyl-asparagine; taurine ↓ Mesobilirubinogen; 2-hydroxycaproic acid; L-urobilin; 6-Hydroxyhexanoic acid; cytidine; dihydrotestosterone; L-histidine; flavin mononucleotide; valyl-serine; 4-acetamidobutanoic acid; (5R)-5-hydroxyhexanoic acid; adenosine; L-targinine; 1,11-undeanedicarboxylic acid; ubiquinone-2; butenylcarnitine; sebacic acid; alanyl-tyrosine	NMIBC vs. Ct Sens = 82% Spec = 95% Accu = 88% AUC = 0.943 MIBC vs. Ct Sens = 90% Spec = 90% Accu = 72% AUC = 0.944
Cheng et al., 2018 [50]	NMIBC vs. Ct: ↑ Dopamine 4-sulfate ↓ Aspartyl-histidine; MG00/1846Z,9Z,12Z,15Z/00 tyrosyl-methionine LG NMIBC vs. Ct: ↑ Heptylmalonic acid ↓ 3-Hydroxy-cis-5-tetradecenoylcarnitine; 6-ketoestriol; β-cortolone; tetrahydrocorticosterone HG vs. LG NMIBC (without haematuria): ↑ 4-2-Aminophenyl-2,4-dioxobutanoic acid; 3-hydroxydecanoyl carnitine; 2-hydroxylauroylcarnitine ↓ 6-Keto-decanoylcarnitine; N-acetyl-4-O-acetylneuraminic acid HG vs. LG NMIBC (with haematuria): ↑ Histidinyl-histidine; N-acetyl-5-methoxykynuramine; L-3-hydroxykynurenine ↓ Indolylacryloylglycine; indoleacrylic acid	NMIBC vs. Ct Training/Discovery Sens = 75% Spec = 79% AUC = 0.857 External validation Sens = 81% Spec = 82% AUC = 0.857 LG NMIBC vs. Ct Training/Discovery Sens = 90% Spec = 79% AUC = 0.938 HG vs. LG NMIBC (without haematuria) Training/Discovery Sens = 75% Spec = 76% AUC = 0.802 HG vs. LG NMIBC (with haematuria) Training/Discovery Sens = 86% Spec = 67% AUC = 0.878
Zhou et al., 2017 [22]	Discovery/Validation set: ↑ Cholesterol ↓ 5-Hydroxyvaleric acid; 3-phosphoglycerate; glycolate	NMIBC vs. Ct Discovery set: Sens = 86% Spec = 86% AUC = 0.875 External set: Sens = 70% Spec = 70% AUC = 0.770 LG BC vs. Ct Discovery set: Sens = 76% Spec = 86% AUC = 0.817 External set: Sens = 63% Spec = 70% AUC = 0.739
Jin et al., 2014 [40]	↑ Succinate; pyruvate; oxoglutarate; carnitine; phosphoenolpyruvate; trimethyllysine; isovalerylcarnitine; octenoylcarnitine; acetyl-CoA ↓ Melatonin; glutarylcarnitine; decanoylcarnitine	NMIBC Accu = 86% MIBC Accu = 50.0%
Huang et al., 2011 [42]	↑ Component I (2-(1-(7-(butyl(ethyl)- amino)heptyloxy)propan-2-yloxy)ethanol) ↓ Carnitine C9:1	LG Sens = 91% Spec = 97% AUC = 0.954

Arrows indicate the direction of change in the first group compared with the second (e.g., ↑ = higher in MIBC than NMIBC). Accu, accuracy; AUC, area under the ROC curve; BC, bladder cancer; Ct, controls; HG, high-grade; LG, low-grade; MIBC, muscle-invasive BC; NMIBC, non–muscle-invasive BC; Sens, sensitivity; Spec, specificity. Information on the dataset or validation approach is reported when available in the original study.

## Data Availability

No data were generated in this study. All data analysed are derived from the cited publications and are summarised in the article and Supplementary Materials.

## Supplementary Materials

The supporting information can be downloaded at https://www.mdpi.com/article/10.3390/ijms27208983/s1.

## Abbreviations

The following abbreviations are used in this manuscript:

AUC	Area under the ROC curve
BC	Bladder cancer
BCAA	Branched-chain amino acids
Ct	Controls
GC-MS	Gas chromatography–mass spectrometry
HG	High-grade
HILIC	Hydrophilic interaction liquid chromatography
LC-MS	Liquid chromatography–mass spectrometry
LG	Low-grade
MALDI-TOF/TOF-MS	Matrix-assisted laser desorption/ionization tandem time-of-flight mass spectrometry
MIBC	Muscle-invasive bladder cancer
MS	Mass spectrometry
NMIBC	Non–muscle-invasive bladder cancer
NMR	Nuclear magnetic resonance
OPLS-DA	Orthogonal partial least squares discriminant analysis
PAGN	Phenylacetylglutamine
PCA	Principal component analysis
PLS-DA	Partial least squares discriminant analysis
RPLC	Reversed-phase liquid chromatography
ROC	Receiver operating characteristic
Sens	Sensitivity
Spec	Specificity
TCA	Tricarboxylic acid cycle
TCE-LDI-MS	Tip-contact extraction–laser desorption/ionisation mass spectrometry
