# Supplementary material for: Urinary Metabolomics in Bladder Cancer: A Scoping Review of Current Evidence and Translation Implications
Source: Int J Mol Sci. 2026 Oct 10;27(20):8983. doi: 10.3390/ijms27208983 (PMC13704385; doi:10.3390/ijms27208983)
Supplement: Supplementary file 1 [file ijms-27-08983-s001.zip › ijms-4613435-supplementary.pdf]

Supplementary material

Urinary Metabolomics in Bladder Cancer: A Scoping Review of Current Evidence and Translation Implications

Ângela Carapito [1,2,*], Filipa Amaro [1,2], Ana Cecília Roque [3,4], Félix Carvalho [1,2], Joana Pinto [1,2,†] and Paula Guedes de Pinho [1,2,*,†]

[1] Associate Laboratory i4HB—Institute for Health and Bioeconomy, University of Porto, 4050-313 Porto, Portugal; famaro@ff.up.pt (F.A.); felixdc@ff.up.pt (F.C.); jipinto@ff.up.pt (J.P.)
[2] UCIBIO—Applied Molecular Biosciences Unit, Laboratory of Toxicology, Faculty of Pharmacy, University of Porto, 4050-313 Porto, Portugal
[3] Associate Laboratory i4HB—Institute for Health and Bioeconomy, NOVA School of Science and Technology, NOVA University of Lisbon, 2829-516 Caparica, Portugal; cecilia.roque@fct.unl.pt
[4] UCIBIO—Applied Molecular Biosciences Unit, Department of Chemistry, NOVA School of Science and Technology, NOVA University of Lisbon, 2829-516 Caparica, Portugal
* Correspondence: angela_c_carapito@hotmail.com (Â.C.); pguedes@ff.up.pt (P.G.d.P.)
† These authors contributed equally to this work.

Table S1. List of urinary metabolomics studies identified through the literature search, summarising cohort characteristics, analytical platforms, and statistical approaches used for BC detection.

Cohort (size)	Analytical platform	Statistical analysis	Ref
BC (n = 50)<br>• MIBC (n = 10), NMIBC (n = 40)<br>Healthy controls (n = 30)	UPLC-QTOF-MS	PCA<br>OPLS-DA<br>Univariate tests (t-test, Mann–Whitney, ANOVA, Kruskal–Wallis)<br>Fold Change analysis<br>ROC analysis<br>Pearson correlation	[27]
Training set:<br>UC (BC + UTUC) (n = 31)<br>• HG (n = 22), LG (n = 9)<br>• Ta-T1 (n = 12), T2-T4 (n = 19)<br>Control (n = 34)<br>Validation set:<br>UC (n = 18)<br>• HG (n = 14), LG (n = 4)<br>• Ta-T1 (n = 10), T2-T4 (n = 8)<br>Control (n = 19)	LC-HRMS	Non-parametric tests<br>FDR correction<br>PCA<br>OPLS-DA<br>ROC analysis	[48]
BC (n = 20)<br>Healthy controls (n = 20)	2D-LC-TQMS (HILIC + RPLC)	PCA<br>PLS-DA	[27]
Training set:<br>BC (n = 70)<br>• Ta (n = 47), T1 (n = 15)	UHPLC-UHRMS	PCA<br>OPLS-DA<br>Mann-Whitney	[28]

Cohort (size)	Analytical platform	Statistical analysis	Ref
• HG (n = 30), LG (n = 38)<br>Controls (n = 70)<br>Validation set:<br>BC (n = 38)<br>• Ta (n = 21), T1 (n = 5), T2 (n = 12)<br>• HG (n = 12), LG (n = 17)<br>Controls (n = 30)		Bonferroni correction<br>ROC analysis<br>RF modelling	
Training set:<br>BC (n = 69)<br>• Primary (n = 41), Recurrent (n = 28)<br>• HG (n = 30), LG (n = 34)<br>• Ta (n = 47), T1 (n = 13), T2 (n = 9)<br>Controls (n = 70)<br>Validation set:<br>BC (n = 30)<br>• Primary (n = 15), Recurrent (n = 15)<br>• HG (n = 11), LG (n = 19)<br>• Ta (n = 21), T1 (n = 6), T2 (n = 3)<br>Controls (n = 30)	^{1}H NMR<br>LDI-MS	PCA<br>OPLS-DA<br>Wilcoxon Rank-sum tests<br>Mann-Whitney<br>Bonferroni correction<br>ROC analysis with RF modelling (AUC>0.7)<br>One-way ANOVA (Tukey's post-hoc testing)	[43]
BC (n = 57)<br>• MIBC (n = 20), NMIBC (n = 37)<br>• HG (n = 28), LG (n = 29)<br>Cancer-free controls (n = 38)	UPLC-MS	PCA<br>OPLS-DA<br>Wilcoxon rank-sum test<br>FDR correction<br>ROC analysis<br>Pearson correlation	[29]

Cohort (size)	Analytical platform	Statistical analysis	Ref
BC (n = 29)<br>• Primary (n = 18), Recurrent (n = 11)<br>• MIBC (n = 12), NMIBC (n = 17)<br>• HG (n = 19), LG (n = 10)<br>• Ta (n = 11), T1 (n = 6), T2 (n = 5), T3 (n = 4), T4 (n = 3)<br>Controls (n = 15)	UPLC-MS	PCA<br>OPLS-DA<br>Pearson correlation<br>RF<br>SVM<br>Boruta analysis	[25]
Training set:<br>BC (n = 51)<br>Controls (n = 49)<br>Validation set:<br>BC (n = 12)<br>Controls (n = 12)	GC×GC-TOF-MS	Wilcoxon rank-sum test<br>Bonferroni correction<br>LOOCV<br>Shapiro-Wilk normality test<br>ROC analysis	[22]
BC (n = 64)<br>Healthy (n = 60)	UHPLC-QTOF-HRMS	One-way ANOVA<br>Shapiro-Wilk normality test<br>Bartlett test of homoscedasticity<br>Wilcox or Welch or Student’s t-test<br>FDR correction<br>ROC analysis<br>PLS-DA<br>PCA<br>HCA<br>Missing value imputation (MVI) algorithms (KNN, SVM, RF, CART…)	[30]
BC (n = 10)	LC-MS/MS	PCA	[31]

Cohort (size)	Analytical platform	Statistical analysis	Ref
Healthy controls (n = 10)		OPLS-DA	
BC (n = 24)<br>Healthy controls (n = 24)	UHPLC-MS	PCA<br>OPLS-DA	[32]
BC (n = 49)<br>Healthy (n = 38)	UPLC-MS/MS<br>MALDI-TOF/TOF<br>TCE-LDI-MS	PCA<br>OPLS-DA<br>Student's t-test	[45]
BC (n = 60)<br>• LG; Ta (n = 20)<br>• HG; Tis/Ta, T1 (n = 20)<br>• HG; T2-T4 (n = 20)<br>Controls (n = 59; hernia)	LC-FT/MS<br>LC-HCT/MS	Chi square tests<br>ROC analysis<br>Odds-ratio analysis	[33]
BC (n = 10)<br>• LG (n = 5), HG (n = 5)<br>KC (n = 10)<br>PCa (n = 10)<br>Healthy (n = 30)	LC-ESI-TOF-MS<br>GC-TQMS	Mann-Whitney<br>Student's t-test<br>Bonferoni correction<br>PCA<br>OPLS-DA	[34]
BC (n = 24; HG/MIBC)<br>Healthy controls (n = 24)	HPLC-TOF-MS<br>^{1}H NMR<br>GC-TQMS	PCA<br>OPLS-DA<br>Mann-Whitney test<br>CV-ANOVA<br>Permutation tests	[24]
BC (n = 40)<br>• HG (n = 20), LG (n = 20)<br>Healthy controls (n = 40)	LC-ESI-TQMS	Student's t-test or Mann-Whitney<br>FDR correction	[35]
Training set:	UPLC-MS/MS	Wilcoxon rank-sum test	[36]

Cohort (size)	Analytical platform	Statistical analysis	Ref
BC (n = 33)<br>• HG (n = 15), LG (n = 18)<br>Healthy controls (n = 44)<br>Validation set:<br>BC (n = 20)<br>• HG (n = 12), LG (n = 8)<br>Controls (n = 26)<br>• Healthy (n = 20)<br>• Benign (n = 6)		FDR correction<br>PCA<br>OPLS-DA<br>ROC analysis	
Training set:<br>BC (n = 50)<br>• HG (n = 33), LG (n = 17)<br>• MIBC (n = 15), NMIBC (n = 35)<br>Healthy controls (n = 35)<br>Validation set:<br>BC (n = 59)<br>• HG (n = 32), LG (n = 27)<br>• MIBC (n = 15), NMIBC (n = 44)<br>Healthy controls (n = 37)	GC-MS	PCA<br>PLS-DA<br>Mann-Whitney<br>FDR correction	[21]
BC (n =46)<br>Healthy individuals (n = 35)	HPLC-FLD	Wilcoxon rank-sum test<br>Pearson correlation<br>Univariate logistic regression	[44]
BC early stage (n = 23)<br>Healthy controls (n = 21)	UPLC-HRMS	PCA<br>OPLS-DA	[37]

Cohort (size)	Analytical platform	Statistical analysis	Ref
		ROC analysis<br>Student's t-test	
Training set:<br>BC (n = 91)<br>Controls (n = 99)<br>• Hernia (n = 68)<br>• UTI/hematuria (n = 31)<br>Validation set:<br>BC (n = 44)<br>Controls (n = 51)<br>• Hernia (n = 14)<br>UTI/haematuria (n = 37)	LC-MS	PCA<br>PLS-DA<br>OPLS-DA<br>ROC analysis	[38]
Training set:<br>BC (n = 138)<br>• Ta (n = 48), T1 (n = 35), T2 (n = 25), T3 (n = 10), T4 (n = 20)<br>Controls (n = 81)<br>Validation set:<br>BC (n = 46)<br>Controls (n = 40)	HPLC-QTOF-MS	PCA<br>OPLS-DA<br>PLS-DA<br>Student's t-test<br>ROC analysis	[39]
Cohort 1:<br>BC (n = 66)<br>• Primary (n = 27), recurrent (n = 39)<br>Non-BC (n = 266)<br>• Haematuria (n = 58)	UHPLC-MS/MS<br>GC-MS	Wilcoxon two-sample test<br>RF<br>ROC analysis	[23]

Cohort (size)	Analytical platform	Statistical analysis	Ref
• History no current (n = 119)<br>• Normal (n = 89)<br>Cohort 2:<br>BC (n = 29)<br>• Primary (n = 12), recurrent (n = 17)<br>Non-BC (n = 79)<br>• Haematuria (n = 20)<br>• History no current (n = 59)			
Training set:<br>BC (n = 38)<br>Non-BC controls (n = 61)<br><br>External validation A:<br>BC (n = 7)<br>Non-BC controls (n = 10)<br><br>External validation B:<br>BC (n = 32)<br>Non-BC controls (n = 31)	GC×GC-TOF-MS<br>LC-MS/MS (for external set B)	PCA<br>PLS-DA<br>OPLS-DA<br>ROC analysis<br>Unpaired t-test and Welch's t-test	[19]
BC (n = 19)<br>KC (n = 25)<br>Healthy (n = 24)	LC-MS (RPLC-MS and HILIC-MS)	PCA<br>OPLS-DA<br>ROC analysis	[46]
BC (n = 27)<br>• LG (n = 21), HG (n = 6)<br>Healthy controls (n = 32)	LC-MS (RPLC-MS and HILIC-MS)	PCA<br>PLS-DA<br>Permutation testing	[41]

Cohort (size)	Analytical platform	Statistical analysis	Ref
		Student's t-test<br>Bonferroni correction<br>ROC analysis	
Group 1:<br>BC (n = 13)<br>• Prior to cystectomy (n = 7)<br>• Prior to TURBT (n = 6)<br>Controls (n = 13)<br>Group 4:<br>BC (n = 9, prior to cystectomy or TURBT)<br>Controls (n = 11)	LC-MS	PLS-DA<br>ROC analysis	[40]
BC (n = 24)<br>Controls (n = 51)	GC-TOF-MS	PCA<br>PLS-DA<br>OPLS-DA<br>Permutation testing<br>ROC analysis	[20]
BC (n = 11)<br>Normal (n = 8)	GC-MS	Student's t-test<br>"Star symbol plotting" (pattern visualization)	[47]
BC (n = 33, NMIBC)<br>• Tis (n = 6), Ta (n = 8), T1 (n = 19)<br>Healthy (n = 37)<br>UTI (n = 31)<br>Bladder stone (n = 2)	^{1}H NMR	Kruskal–Wallis<br>Mann–Whitney<br>Bonferroni correction	[42]

^{1}H NMR, proton nuclear magnetic resonance; ANOVA, analysis of variance; AUC, area under the curve; BC, bladder cancer; CART, classification and regression tree; CIS, carcinoma *in-situ*; CV, cross-validation; ESI, electrospray ionization; FDR, false discovery rate; GC, gas chromatography; GC–MS, gas chromatography–mass spectrometry; GC×GC–TOF–MS, two-dimensional gas chromatography coupled with time-of-flight mass spectrometry; HCA, hierarchical cluster analysis; HG, high-grade; HILIC, hydrophilic

interaction liquid chromatography; HPLC, high-performance liquid chromatography; KC – kidney cancer; LC–FTMS, liquid chromatography coupled with Fourier transform mass spectrometry; LC–HCTMS, liquid chromatography–ion trap mass spectrometry; LC–HRMS, liquid chromatography–high-resolution mass spectrometry; LC–MS/MS, liquid chromatography–tandem mass spectrometry; LG, low-grade; LOOCV, leave-one-out cross-validation; MALDI, matrix-assisted laser desorption/ionization; MIBC, muscle-invasive bladder cancer; MVI, missing value imputation; NMIBC, non-muscle-invasive bladder cancer; OPLS–DA, orthogonal partial least-squares discriminant analysis; PCA, principal component analysis; PCa, prostate cancer; PLS–DA, partial least-squares discriminant analysis; RF, random forest; RPLC, reversed-phase liquid chromatography; SVM, support vector machine; TCE–LDI–MS, tip-contact extraction–laser desorption/ionization mass spectrometry; 2D–LC–TQMS, two-dimensional liquid chromatography coupled with triple quadrupole mass spectrometry; UC, urothelial carcinoma; UHPLC–UHRMS, ultra-high-performance liquid chromatography–ultra-high-resolution mass spectrometry; UPLC–QTOF–MS, ultra-performance liquid chromatography coupled with quadrupole time-of-flight mass spectrometry; UTI, urinary tract infection; UTUC, upper tract urothelial carcinoma; VIP, variable importance in projection.
